# Active Assistance Technology for Health-Related Behavior Change: An Interdisciplinary Review

**DOI:** 10.2196/jmir.1893

**Published:** 2012-06-14

**Authors:** Catriona M Kennedy, John Powell, Thomas H Payne, John Ainsworth, Alan Boyd, Iain Buchan

**Affiliations:** ^1^School of Community-Based MedicineUniversity of ManchesterManchesterUnited Kingdom; ^2^School of Computer ScienceUniversity of BirminghamBirminghamUnited Kingdom; ^3^Division of Health SciencesUniversity of WarwickWarwickUnited Kingdom; ^4^UW MedicineUniversity of WashingtonSeattle, WAUnited States; ^5^Health Policy and Management GroupManchester Business SchoolManchesterUnited Kingdom

**Keywords:** Behavior change, consumer health informatics, health communication, health promotion, personalization

## Abstract

**Background:**

Information technology can help individuals to change their health behaviors. This is due to its potential for dynamic and unbiased information processing enabling users to monitor their own progress and be informed about risks and opportunities specific to evolving contexts and motivations. However, in many behavior change interventions, information technology is underused by treating it as a passive medium focused on efficient transmission of information and a positive user experience.

**Objective:**

To conduct an interdisciplinary literature review to determine the extent to which the active technological capabilities of dynamic and adaptive information processing are being applied in behavior change interventions and to identify their role in these interventions.

**Methods:**

We defined key categories of active technology such as semantic information processing, pattern recognition, and adaptation. We conducted the literature search using keywords derived from the categories and included studies that indicated a significant role for an active technology in health-related behavior change. In the data extraction, we looked specifically for the following technology roles: (1) dynamic adaptive tailoring of messages depending on context, (2) interactive education, (3) support for client self-monitoring of behavior change progress, and (4) novel ways in which interventions are grounded in behavior change theories using active technology.

**Results:**

The search returned 228 potentially relevant articles, of which 41 satisfied the inclusion criteria. We found that significant research was focused on dialog systems, embodied conversational agents, and activity recognition. The most covered health topic was physical activity. The majority of the studies were early-stage research. Only 6 were randomized controlled trials, of which 4 were positive for behavior change and 5 were positive for acceptability. Empathy and relational behavior were significant research themes in dialog systems for behavior change, with many pilot studies showing a preference for those features. We found few studies that focused on interactive education (3 studies) and self-monitoring (2 studies). Some recent research is emerging in dynamic tailoring (15 studies) and theoretically grounded ontologies for automated semantic processing (4 studies).

**Conclusions:**

The potential capabilities and risks of active assistance technologies are not being fully explored in most current behavior change research. Designers of health behavior interventions need to consider the relevant informatics methods and algorithms more fully. There is also a need to analyze the possibilities that can result from interaction between different technology components. This requires deep interdisciplinary collaboration, for example, between health psychology, computer science, health informatics, cognitive science, and educational methodology.

## Introduction

Prevention, early intervention, and self-care are priorities for most health care systems around the world. Policy makers cannot, however, address these priorities solely through conventional clinical means. This is because citizens must make sustained health behavior changes, which are largely beyond the reach of the clinic [1].

Information technology has the potential to support behavior change [2]. However, in many behavior change interventions, technology is used as only a passive medium, where the main purpose is efficiency, communication, or a positive user experience. For example, an interactive website might provide different types of multimedia or a discussion forum, but the health information may be static. Even if the health content is tailored to a particular audience, the tailoring is determined a priori. Users navigate the website in the same way as they would navigate tailored print material, albeit often with greater ease. Processing of health content is not automated.

Furthermore, many interventional studies do not identify which *aspects *of technology contributed to the observed outcomes; the technologies are simply named in a general way (eg, mobile phones [3]). Recent reviews on technology-supported interventions [4-7] also did not explore the semantic processing potential of the technology. For example, Enwald and Huotari [5] defined “second generation” as “interactive media” and “third generation” as the use of “mobile and remote devices.”

In this paper we aim to review research on the *active *aspects of technologies to assist health behavior change. Our objectives were (1) to define key technological capabilities involving active information processing, (2) to determine through a literature review where these technologies are used in behavior change interventions or where they are clearly recognized as important for interventions, (3) to identify emerging themes involving novel uses of active technology that are cross-disciplinary, and (4) to identify any gaps in the research and propose future directions.

### Definition: Active Assistance

We define active assistance technology as any technology involving *automated processing of health or behavior change information *that is *ongoing as the user interacts with the technology*. In other words, the technology continues to process the health-related information during interactive use and may adapt its responses. This contrasts with passive information technology such as storage devices, videos, and website design. It also contrasts with interactive systems that do not process information related to health or behavior change. For example, an interactive system can process user choices on presentation format (eg, video or text) and adapt to these choices during a session. However, this is not active assistance because the responses are not related to the semantic content of the health messages, only to their formatting.

In this way, the concept of active assistance draws attention to the distinction between semantic and nonsemantic information processing during an interactive session. This is important, because semantic processing entails a degree of delegation of health decision making to an automated system, which can free up human specialists. It also has more serious consequences if incorrect.

Furthermore, active assistance takes place in an environment in which citizens and experts participate actively in the behavior change intervention (combining push and pull). In other words, the role of the technology is not merely to deliver a fully expert-led intervention where users follow instructions with minimal understanding. Instead, the technology helps users to reflect and learn about the obstacles to successful behavior change. A desirable feature is that users should feel they have ownership or control of the intervention [8].

A concept related to active assistance is persuasive technology, which is “designed to change attitudes or behaviors of users through persuasion and social influence” [9]. Although many aspects of persuasion are relevant to active assistance, persuasive technology is not necessarily active in the way that we are defining it; passive techniques such as visualization and website design may suffice. However, some aspects of persuasion would benefit from active technology. In particular, the potential of the technology for unbiased information processing can help with self-monitoring and correction of inaccurate health beliefs, both of which are required in the persuasion model [10,11]. Similarly, many behavior change theories such as the transtheoretical model [12], social cognitive theory[13-15], and theory of planned behavior [16] imply the need for health education, accurate beliefs, and self-monitoring to effect behavior change and sustain it over the long term. Independently of persuasion and self-monitoring, automated information processing can also overcome some of the problems of self-report in measuring behavior change such as adherence [17].

In the context of these requirements, the following are key examples of active assistance technology that can support behavior change. The technologies may be used together or independently.

#### Automated Reasoning Using an Explicit Knowledge Representation

An example of this is that an interactive system might use knowledge-based reasoning about the user’s health and circumstances to determine how its responses should be tailored further to the particular individual. Since this process is dynamic, there is more potential for delivery of messages that are tailored to the user’s current environment and state of motivation than would be the case for static tailoring. Similarly, health education can involve answering specific health-related questions, on demand, using inference about what-if scenarios that the user wants to know about (nor necessarily personalized). Examples might include mobile or Web interfaces with dynamic personalization, intelligent reminding, natural-language dialog, or health-related games with an automated player.

#### Automated Data Collection With Pattern Recognition (Smart Sensing)

Automated sensing can overcome the limits of self-reported online diaries, which depend on memory. Recognition of patterns in online interactions, physical activities, or physiological states can provide useful self-monitoring information for users who are attempting to change their behavior, provided that it is displayed in a user-friendly way. This goes beyond the capability of automated reasoning because the system can acquire data and recognize events automatically without manual data input.

#### Automated Adaptation Over Time

Adaptation occurs in response to emerging patterns and contexts. This goes beyond automated reasoning and automated data collection because the system adapts its methods and decisions according to patterns that it has recognized. For example, an interactive system might learn to predict a user’s state of motivation based on his or her responses to prompts (without any additional sensing).

In each case, the algorithms need to be informed by health-related knowledge, either explicitly as a formal representation or implicitly in the form of assumptions built into their design.

### Background: Related Reviews

Some previous literature reviews have addressed topics related to active assistance.

Webb et al [2] conducted a systematic review and meta-analysis of the effectiveness of Internet-based interventions. The review found that interventions grounded in behavior change theories are associated with increased effectiveness, with the theory of planned behavior having the largest positive effect. The review used a taxonomy of behavior change techniques and found that using a combination of these techniques tended to be more effective than using only one. Effectiveness was also increased by communication methods such as instant messaging. Although the review did not consider the role of active semantic processing, the findings that a combination of techniques is more effective suggest an increased necessity for such automated processing due to the added complexity of interventions that combine more than one technique.

Fry and Neff [18] reviewed the effectiveness of interventions using automated prompts and reminders, finding that more than half of those (11 out of 19 reviewed) showed positive results. The findings suggest that tailoring of prompts and making them more interactive can make them more effective. However, the review did not specify the role of the technology in determining the timing and content of prompts: whether these are manually designed in advance, or whether they can be adapted using automated methods.

Lustria et al [4] defined second-generation interventions as those that are tailored to individuals. The definition did not specify whether the tailoring is an ongoing automated adaptation process that happens during continued user interaction. However, the review has some interesting findings. Many tailored interventions have components for developing self-regulatory skills and self-monitoring. For example, the user can often keep online diaries and send them to an expert for analysis, who then provides feedback. Automation of some of the semantic analysis steps in this process would reduce the need for human expert involvement, thereby reducing costs. Similarly, Revere and Dunbar [19] conducted an earlier review (1996–1999), finding that tailored interventions tended to be effective but not enough evidence that tailoring was superior to generic interventions. The review mentioned the possibility of dynamic tailoring but did not focus on the distinction between dynamic and offline computer tailoring.

Bickmore and Giorgino [20] reviewed the technologies used in dialog systems and presented some examples that have been evaluated as effective. Similarly, Corkrey and Parkinson [21] reviewed interactive voice response systems from 1989 to 2000, finding a growing research area but few applications at that time.

For the purpose of building personalized health models, Fernandez-Luque et al [22] reviewed the research on extracting content from social networking sites (eg, blogs, forums, or search patterns). These techniques have relevance in behavior change interventions but are still at an early stage and present many ethical and technical challenges.

There have been no comprehensive reviews of active assistance technologies in health-related behavior change.

## Methods

We conducted the literature review in accordance with the guidelines of the PRISMA statement [23] that are relevant to our objectives.

### Keyword Search and Databases

We used the following strategy: <technology-related keywords> AND <health psychology-related keywords> AND health AND “behavior change”.

Technology- and psychology-related keywords were combinations of the following: [automated OR technology OR Internet OR “mobile phone” OR intelligent OR computer-based OR interactive OR agent-based OR adaptive OR “context-aware” OR “machine learning” OR “pattern recognition” OR robotic OR “virtual reality” OR semantic OR “knowledge-based” OR “decision support” OR ontology OR dialog OR “natural language”] AND [assistance OR intervention OR personalization OR persuasion OR adherence OR compliance OR motivation OR affective OR emotion OR reminder OR prompt].

We conducted some preliminary searches on a wide range of databases including CINAHL, EMBASE, Inspec, ISI Web of Science, PsycINFO, and ScienceDirect. However, we found that Google Scholar had a wide enough coverage to allow it be used instead of these databases. This is consistent with recent empirical studies such as those of Howland et al [24] and Walters [25]. Therefore, we decided to use only Google Scholar and PubMed. The date range was January 2005 to January 2012. Only articles written in English were included.

### Inclusion and Exclusion Criteria

We used the definition of active assistance above. The studies should have included at least one of the three active assistance technologies listed and have been intended for interactive use by clients or patients attempting to change their behavior. Studies may have described a new technology or design to be used in behavior change interventions, or they may have reported an evaluation of an actual intervention using the technology. Qualitative studies are necessary to evaluate usability and acceptability of active technology. Similarly, prototypes and works in progress help to provide an overview of the current research concepts and their maturity.

We excluded the following kinds of study: (1) interventions merely delivered using the Internet, CD-ROM, or other medium, where the technology only facilitates transmission of information from expert to user, (2) feedback, in which there is no automated processing of personal health-related information—for example, receiving emails from a human counselor would be excluded, (3) tailored-offline interventions, in which the computer processing is used by health professionals to tailor the intervention before or after the user interacts with the technology—this is the case where the semantic content processing is not part of the interactivity with the client (although a health professional may have interactive access), (4) simple data collection or preprogrammed reminding without any pattern recognition or inference (eg, pedometers); interventions where the semantic content of reminder messages were configured by the client were also excluded for this reason—for example, the planning tool of Soureti et al [26] is a passive technology and therefore excluded, (5) fixed games or simulations, in which there is no automated player with semantic processing capability, and (6) general guidelines or research roadmaps in which different options are discussed.

### Data Extraction

We divided studies into the following categories: (1) quantitative and qualitative evaluations of interventions using active technology, (2) pilot studies of new technology, and (3) prototypes or designs that are being developed or tested.

For each study, we asked the following questions. What kind of active technology was used? Was it effective? What was the role of the technology in the intervention? Was it theoretically grounded?

#### Active Technology Types

We looked for one or more of the following types of automated content processing, based on capabilities of the active assistance technologies outlined above: (1) automated data collection with pattern recognition, (2) context-sensitive alerts, reminders, and recommendations, (3) knowledge-based reasoning or inference (semantic representation, ontology, decision support, decision algorithm, and automated planning), (4) dialog systems with natural-language processing, (5) simulation or game with an intelligent agent, and (6) online adaptation to build user models and personalization (adaptive websites or interfaces, and user profiling).

In addition to our predefined categories, we identified new technology themes and author keywords describing the technology.

#### Effectiveness Evaluations

For those studies with evaluations of effectiveness, we asked the following questions. First, what was being evaluated? This could be acceptability or usability (self-reported positive or negative attitude); treatment adherence or technology engagement (observed); self-reported behavior change; or objectively measured behavior change (eg, step counts). Second, what method of evaluation was used (eg, randomized controlled trial [RCT] or qualitative study)? Third, were findings summarized, to give an indication of the maturity of the technology, and any advantages or new problems that it introduces?

#### Role of Active Technology in Interventions

It is important to understand the role of the active technology in the intervention.

We used the following three functions (defined above): (1) dynamic tailoring, (2) interactive education to support participations in their own care and disease prevention, and (3) support for self-monitoring in a way that overcomes biases of self-report.

In particular, we looked for an association of an active technology type with a purpose. For example, pattern recognition and context awareness may be used to support dynamic tailoring. Similarly, for unbiased self-monitoring, the technology needs to provide automated data collection, pattern recognition, and representation of the results in a visual format that can be easily understood.

#### Theoretical Grounding

We included here any behavior change theories mentioned by the authors as having a role in the technology design. In addition, we asked whether the study proposed any novel ways of connecting active technology with behavior change theories, and whether the active technology allows new possibilities that would not be available with static technology.

## Results

Following a review of title and abstracts, the search identified 228 potentially relevant articles. Of these, 41 satisfied the inclusion criteria after a full-text review. Table 1 lists the data extraction contents for intervention and active technology themes [27-67], along with any effectiveness evaluations. Table 2 [27-67] shows the extent to which our previously defined technology roles appeared in the studies (dynamic tailoring, interactive education, and self-monitoring support). Also included are any behavior change theories informing the technology design or usage.

In Table 1, we used the following notation to give an approximate summary of evidence for studies with effectiveness evaluations: <weight of study>: <effect>. Weight of study was scored as 5 (RCT with at least one objective measure, long-term), 4 (RCT with at least one objective measure, short-term), 3 (RCT with self-report only, long-term), 2 (RCT with self-report only, short-term), or 1 (qualitative or pilot study). Effect was scored as + (positive), (negative), or +/– (mixed or insignificant).

For example, a study with objective measures over the long term, but not showing a significant effect, would be summarized as 5: +/–. We used the same summarized notation if some measures were positive and others negative or insignificant. Details are in the findings column.

**Table 1:** Technology themes, study types, and main findings.

Reference and project or intervention name	Health topic / study population	Technology themes	Type of study	Main findings	Evidence summary (if applicable)^a^
Ananthanarayan & Siek 2010 [27] (HealthSense)	Obesity / children	Wearable computing, “6th sense;” actionable feedback.	Design of prototype to support children’s motivation for exercise and for self-monitoring.	Not an empirical study.	Not applicable
Arteaga et al 2009 [28]	Obesity / teenagers	Motivational agent (mobile phone games).	Design of prototype to motivate exercise based on personality type.	Not an empirical study.	Not applicable
Bickmore & Picard 2005 [29] (FitTrack)	Physical activity / healthy adults	ECA^b^: relational agent.	RCT^c ^(n = 101; 30 days). Measures: acceptability (self-report) + PA^d ^(pedometer). Groups: relational agent, nonrelational agent and control.	Positive acceptance; increased PA during intervention but reduced PA after follow-up. Relational agent more liked. Dialog repetitiveness annoying.	4: +/–
Bickmore et al 2005 [30] (FitTrack)	PA / older adults	ECA: relational agent.	RCT (n = 21; 2 months), to test acceptability (usage history) + PA (pedometer) + loneliness (self-report). Groups: relational vs control (usual care).	Positive acceptance and significant increase in PA during intervention. No significant decrease in loneliness.	4: +/–
Bickmore & Sidner 2006 [31]	General behavior change / adults	Making dialog more robust by linking with ontologies for behavior change theories (TTM^e^, MI^f^).	Prototype.	Not an empirical study.	Not applicable
Bickmore et al 2009 [32]	Physical activity / adults (male students)	Context awareness of mobile PA monitor + ECA (relational agent).	Pilot study (n = 8): test whether agent context awareness promotes social bonding (acceptance). Effectiveness: does it promote walking?	Some positive acceptance but less actual walking in context-aware condition.	1: +/–
Bickmore et al 2009 [33]	Compliance / low health-literacy patients (hospital discharge)	ECA: virtual nurse with relational behaviors and empathy.	Self-report usability tests: 2 tests: nonpatients (n = 30) + patients (n = 19) with 47% low literacy. Both groups tested with relational vs nonrelational agent.	Both tests: relational preferred. Overall ECA acceptance. ECA allows more time and sense of control than human face-to-face communication.	1: +
Bickmore et al 2010 [34]	Medication adherence, PA / schizophrenia patients	ECA: simple concrete communication. Authors counter ethical criticism of ECA for mental health.	Pilot evaluation (n = 20; 31 days) to test acceptability (self-report) + adherence + PA (no control).	Positive acceptance. Adherence + PA high. ECA provides simplified conversation, less confusing than human face-to-face.	1: +
Bickmore et al 2011 [35]	2 domains: exercise and diet / adults	Semantic ontology for behaviors and theories (TTM, MI); semantic models of user, data, and intervention.	Qualitative study (n = 8) on acceptability of ECA health counselor based on reusable ontology.	Positive acceptance, but limited evaluation.	1: +
Bickmore et al 2010 [36]	Physical activity / adults	ECA: promoting long-term use; avoid repetitive dialog. Introduce variability + storytelling.	2 RCTs: 1. Variability (n = 24, 100 days); variable vs nonvariable; 2. Story(n = 26, 30 days): first-person story vs third-person story. Measures: usage + step count + self-reported satisfaction.	1. Variability: more system usage, but less exercise. 2. Story: first person had more usage than third person, but less exercise. Self-reported satisfaction high for test conditions.	4: +/–
Bieber et al 2009 [37] (DiaTrace)	Physical activity / adults	Mobile phone as sensor for activities and calorie estimate.	Prototype.	Not an empirical study.	Not applicable
Buttussi & Chittaro 2008 [38] (MOPET)	Physical activity / adults	ECA; context-aware sensing; user model.	Prototype.	Initial qualitative evaluation positive (n = 12).	1: +
Consolvo et al 2008 [39] (UbiFit)	Physical activity / adults	Graphic display with “garden” metaphor; mobile sensing device with inference; interactive app (edit or add to journal).	RCT: 3-month field experiment (n = 28): full system (10) vs no mobile sensing device (9) vs no display (9). Measures: (1) sensed activities + self-report; (2) qualitative analysis on user experience.	System with display led to more exercise than without display. User experience positive: more self-awareness, which motivated exercise.	4: +
De Rosis et al 2006 [40]	Diet / adults	ECA: recognize user’s emotional state, social attitude, and TTM stage during dialog; dynamically update user model during dialog.	Prototype: raters label emotional states, TTM stages, and social attitudes in test dialogs (WOZ^g ^and corpus).	Labeling of emotions by raters used to guide design of dialog system.	Not an intervention evaluation
Farzanfar et al 2007 [41]	Treatment adherence, suicide prevention / depressed adults	Telephone agent: monitoring + self-care management.	Preliminary qualitative trial (n = 15), 4 weeks. Modifications made in response.	Dialog was helpful for adherence, but sounded artificial and insensitive, particularly in suicide risk. Users prefer more human-like agent with empathy and understanding of serious issues. (For suicide, hotline preferred). Authors’ conclusion: anthropomorphism is not valid (people do not attribute human qualities to machines—only in metaphor).	1: +/–
Hakulinen et al 2008 [42] (COMPANIONS project)	PA / adults	Mobile companion; semantic ontology of user environment for PA planning.	Prototype.	Not an empirical study.	Not applicable
Hartmann et al 2007 [43]	Improve patient questions to physician / adults with asthma	Educational website to suggest questions, encourage patient involvement in care, prevent more serious illness.	Pilot study: (n = 37) record usage experiences.	Positive self-report: (1) improved relations, (2) more active involvement.	1: +
Hayes et al 2009 [44]	Medication adherence / older adults	Instrumented pillbox, home sensors.	Pilot study (n = 10): effectiveness of context awareness on adherence. Test phases (same group): no-prompt, time-based, context-aware prompt.	Initial evaluation: positive for context-aware phase.	1: +
Jin 2010 [45]	Stress management / college students	Education-entertainment / health belief, self-efficacy; educational interactive test (game) for responses to stress scenarios. Agent gives educational messages.	RCT: (n = 60). Effectiveness of virtual agent on student’s intent/mood. Interactive test with agent (test) vs no agent (control) vs no test (true control).	Positive self-report on enjoyment and educational value for agent condition. Interactive test improves stress management self-efficacy (over true control, without test).	2: +
Kaipainen et al 2011 [46] (HealthPGS)	General health decisions / adults	Health Personal Guidance System: guide user through day-to-day choices in ecosystem. Virtual individual: maintains user profile and context; HealthGuide: planning, context-aware messages. Personal Guidance System Mall: services all in one place.	Prototype.	Not an empirical study.	Not applicable
Klein et al 2011 [47] (eMate)	Adherence / diabetic patients	Automated reasoning based on COMBI^h ^model (combines different theories) ensures dynamic tailored messages depending on user’s context and state of mind.	Prototype: computational model of behavior change (mobile + website).	Not an empirical study.	Not applicable
Konovalov et al 2010 [48] (GATE)	Mental health promotion / military service personnel	Blog analysis to understand moods and emotions (combat experience): GATE algorithm + ontology.	Design and pilot study for technology: compare algorithm with expert opinion.	Precision of algorithm: 0.9, recall: 0.75; *F *score: 0.82.	Not an intervention evaluation.
Lee et al 2010 [49]	Health promotion / older adults	Telehealth: action-based behavior model (1) increase user’s awareness of health, (2) set goals, (3) educate user in how to achieve goal, (4) remind, (5) reward + assess.	Design: overcome limits of sensing only; need high-level assessment information with models of persuasion to determine whether behavior changed.	Not an empirical study.	Not applicable
Levin & Levin 2006 [50]	Pain management / adults	Ecological momentary assessment, detect unexpected errors in dialog.	Feasibility study: evaluate interactive voice response system dialog for health and behavior monitoring. Feasibility study for pain monitoring voice diary (n = 24). 171 dialog sessions.	Accuracy of voice recognition: 98%. Dialog efficiency increased with user experience.	1: +
Lisetti & Wagner 2008 [51] (ABLE)	Mental health promotion / adolescent	ECA companions.	Design: ECA companion to act as MI counselor.	Not an empirical study.	Not applicable
Looije et al 2010 [52] (SuperAssist)	Adherence / older adults	ECA (robot cat), MI, persuasion.	Pilot study (n = 24): physical ECA (n = 12) vs virtual (n = 12). Each group experienced text, social ECA, and nonsocial ECA.	90% acceptance. Social ECA preferred over nonsocial ECA; half preferred text interface over social ECA (“conscientious” personality type). Virtual ECA more “empathic” than physical.	1: +
Maier et al 2010 [53] (SEMPER)	Work-related disorders and alcohol / adults	Semantic Web portal; semantic search.	Prototype.	Not an empirical study.	Not applicable
Mazzotta et al 2007 [54] (PORTIA)	Healthy eating / adults	Persuasion agent: tailoring of messages based on inferred personality traits and likely motivations of user.	Prototype of dialog design, based on corpus analysis of persuasive dialogs produced by participants in role-playing scenarios.	Corpus analysis found that persuasion is most often based on nonrational arguments and positive framing.	Not an intervention evaluation
Munguia Tapia 2008 [55]	Obesity / adults	Sensors and algorithms for activity recognition and energy expenditure estimate.	Prototype.	Activity recognition most accurate if simple examples are given; high variability is difficult (eg, housework). Energy estimate more accurate for simple activities and with multiple body sensors.	Not an intervention evaluation
Nguyen & Masthoff 2008 [56]	General behavior change / adults	Persuasive dialog, MI.	Acceptability test (n = 41): is MI dialog more persuasive than argumentation? Questionnaire + qualitative analysis in comments.	Self-report positive: persuasiveness, likeability scores higher for MI than for 2 types of argumentation.	1: +
Oddsson et al 2009 [57] (SKOTEE)	Adherence / adults	Robotic assistance for intelligent reminding and companionship.	Design.	Not an empirical study.	Not applicable
Op den Akker et al 2011 [58]	PA / adults	Software agent for smart phone: use machine learning to develop user model. Tailor messages to user history and current context.	Prototype.	Not an empirical study.	Not applicable
Rojas-Barahona & Giorgino 2009 [59] (AdaRTE)	General behavior change / adults	Framework for health dialog.	Design.	Not an empirical study.	Not applicable
Smith et al 2008 [60]	Healthy lifestyle / adults	ECA; collaborative planning, update planned activities through ongoing dialog.	Prototype with technical evaluation.	Approach is feasible, although dialog error rate is still high.	Not applicable
Sorbi et al 2007 [61]	Migraine attack prevention / adults	PDA^i ^+ coaching. Response behaviors to precursors of migraine. Ecological momentary intervention experience sampling: randomized calls overcome memory bias. Tailored messages depending on current experience.	Pilot study (n = 5): feasibility and user acceptance.	Positive acceptance but too many calls are annoying. Technical problems: data loss due to buildings.	1: +/–
Spring et al 2010 [62] (Make Better Choices–MBC)	Obesity / adults	PDA: find optimal advice for exercise; goal thermometers; “in the moment” decision support/multiple theories, including self-regulation.Study design.	Study design.	Design of a trial only.	Not applicable
Tiwari et al 2011 [63]	Adherence / older adults	Robotic assistance, dialog.	Prototype development using grounded theory participatory design.	Emerging themes: usability, empowerment, collaboration, and safety: used as requirements for dialog design.	Not applicable
Turunen et al 2011 [64] (COMPANIONS project)	Health and fitness / adults	Home and mobile health and fitness companion.	Pilot study (n = 20): feasibility of complex dialogues in home and mobile scenarios.	System behaves robustly in realistic experimental scenarios, but word error rates are still high.	1 +
Uribe et al 2011 [65]	Adherence general	Reminders based on inferred mental state; user modeling using ontologies.	Prototype.	Not an empirical study.	Not applicable
van der Putten et al 2011 [66] (SERA projet–Social Engagement with Robots and Agents)	PA / older adults	Social robot; health advisor.	Pilot study(n = 6). Video recording of interactions in homes. Iteratively modify setup based on results of previous session.	3 iterations, variable interactions, and satisfaction reports. Positive for motivation but some frustration over lack of control of dialog and too much time taken up.	1: +/–
Watson et al 2012 [67]	PA / overweight adults	ECA: virtual coach	RCT (n = 70; 12 weeks); primary measure: step count; secondary: weight + self-reported satisfaction, self-efficacy, PA recall, and PA stage of change. Groups: virtual coach vs control (no coach: website + pedometer only).	Average step count for intervention group remained constant over 12 weeks while control group dropped. Repeated measures analysis of variance showed significant difference in step count change between intervention and control. No significant difference in secondary measures; acceptance mixed.	4: +/–

^a ^<weight of study>: <effect>: weight of study was scored as 5 (randomized controlled trial [RCT] with at least one objective measure, long-term), 4 (RCT with at least one objective measure, short-term), 3 (RCT with self-report only, long-term), 2 (RCT with self-report only, short-term), or 1 (qualitative or pilot study). Effect was scored as + (positive), (negative), or +/– (mixed or insignificant).

^b ^Embodied conversational agent.

^c ^Randomized controlled trial.

^d ^Physical activity.

^e ^Transtheoretical model.

^f ^Motivational interviewing.

^g ^Wizard of Oz study, where humans pretend to be dialog agents to understand the likely responses to an automated agent.

^h ^Computerized behavior intervention.

^i ^Personal digital assistant.

**Table 2:** Active technology role and theoretical grounding.

Reference and project or intervention name	Active technology type	Dynamic tailoring	Interactive education	Self-monitoring	Theoretical grounding
Ananthanarayan & Siek 2010 [27] (HealthSense)	Inference; pattern recognition	Not specified	Yes, but details not given	Yes; provide awareness of physical activity	General awareness only; no specific theory mentioned
Arteaga et al 2009 [28]	Dialog; pattern recognition	Not specified. Static tailoring only	No	Not specified	Big 5 personality theory; technology acceptance model; theory of planned behavior, theory of meaning behavior
Bickmore & Picard 2005 [29] (FitTrack)	Dialog	Not specified	No; passive educational content only	Very basic, pedometer steps only	Relational agents
Bickmore et al 2005 [30] (FitTrack)	Dialog	Not specified	No; passive educational content only	Progress charts only	Relational agents
Bickmore & Sidner 2006 [31]	Inference; dialog	Not specified, but possible	No	Progress charts only	TTM^a^, MI^b^: link with agent reasoning and ontology
Bickmore et al 2009 [32]	Pattern recognition	Not specified in detail	No	No	Relational agents
Bickmore et al 2009 [33]	Dialog	Not specified in detail, only mentioned as a property of dialog systems in general	Yes, support low health-literacy patients	No	Relational agents
Bickmore et al 2010 [34]	Dialog	Not specified in detail	No	Not considered usable by schizophrenia patients	Relational agents
Bickmore et al 2011 [35]	Inference; dialog; user models	Not specified; fixed tailoring only mentioned	No, but mentioned in a generic way	No	TTM, MI encoded in ontology for agent reasoning and user model
Bickmore et al 2010 [36]	Dialog	Not specified	No	Charts only	Relational agents
Bieber et al 2009 [37] (DiaTrace)	Physical activity recognition, mobile phones	Not specified	No	No	Not mentioned
Buttussi & Chittaro 2008 [38] (MOPET)	Pattern recognition; adaptation; user model	Yes, due to context awareness	No	Not mentioned, but possible	Not mentioned
Consolvo et al 2008 [39] (UbiFit)	Activity recognition; inference	Not specified	No	Yes; visual display	No
De Rosis et al 2006 [40]	Dialog; user modeling, adaptation	Yes, due to adaptation	No	No	TTM
Farzanfar et al 2007 [41]	Dialog; pillbox sensors + adherence data analysis—linked to dialog system	Not specified, although possible	Yes, telephone instructions but limited interactivity	No	Self-efficacy theory, MI
Hakulinen et al 2008 [42] (COMPANIONS project)	Dialog; automated planning; knowledge-based inference	Not specified, although possible	No	No	Not mentioned
Hartmann et al 2007 [43]	Inference: evidence-based decision rules	Not specified in detail, but possible	Yes, but limited	No	No
Hayes et al 2009 [44]	Context-aware reminders; activity recognition; rule-based inference	Yes, decision to prompt based on recognized activity pattern	No	Not mentioned, but possible to include	Not mentioned
Jin 2010 [45]	Virtual agent in game	Not specified	Yes, education-entertainment	No	Health belief model, self-efficacy
Kaipainen et al 2011 [46]	Context awareness, pattern recognition, inference, planning, user modeling	Yes, messages tailored to changing context of user	Not a main focus	Not mentioned, but possible to include	Hybrid approach including self-efficacy and social influence
Klein et al 2011 [47] (eMate)	Knowledge-based reasoning; user models	Yes, automated reasoning based on COMBI^c ^model ensures dynamic tailored messages depending on user’s context and state of mind	No	No	COMBI model includes aspects of TTM, health belief model, social cognitive theory, self-regulation theories, attitude formation theory, and relapse prevention model; interaction based on MI
Konovalov et al 2010 [48]	Pattern recognition; inference	No, but could be used in an intervention with dynamic tailoring	No	No	No
Lee et al 2010 [49]	Pattern recognition; user modeling (profiling), including mental states	Not specified in detail, but planned	Not specified, but planned	Not specified, but planned	Action-based behavior model: (1) increase user’s awareness of health; (2) set goals; (3) educate user in how to achieve goal; (4) remind; (5) reward + assess
Levin & Levin 2006 [50]	Voice recognition; semantic representation; dialog	Not specified, but personalization of dialog possible	No	No	No
Lisetti & Wagner 2008 [51] (ABLE)	Dialog system considered	Not specified, but possible	No	No	MI
Looije et al 2010 [52] (SuperAssist)	Dialog	Not specified, but possible	No	No	MI
Maier et al 2010 [53] (SEMPER)	Text mining; ontologies; machine learning; semantic search	Yes, personalized search results based on user profile built automatically	Yes, information portal	No	MI
Mazzotta et al 2007 [54] (PORTIA)	Dialog, user model	Yes, tailoring of persuasion messages based on inferred personality traits and likely motivations of user	No	No	Persuasion theories, argumentation
Munguia Tapia 2008 [55]	Activity recognition; energy estimate	No, but possible in an intervention	No	No, but possible in an intervention	No
Nguyen & Masthoff 2008 [56]	Dialog	Not specified	No	No	MI-based dialog design
Oddsson et al 2009 [57] (SKOTEE)	Intelligent reminding	Yes, part of robotic companion	No	Not mentioned, but possible to include	No
Op den Akker et al 2011 [58]	Pattern recognition, machine learning, context awareness, user modeling	Yes, messages are tailored based on user model and context	No	Not mentioned	No
Rojas-Barahona & Giorgino 2009 [59] (AdaRTE)	Dialog; adaptation	Yes, dialog can be adapted according to patient answers	No	No	No
Smith et al 2008 [60] (COMPANIONS)	Dialog control; inference; automated planning	Yes, update planned activities through ongoing dialog	No	No	No
Sorbi et al 2007 [61]	Adaptation, automated personalized feedback	Yes, tailored messages depending on current experience	No	No	No
Spring et al 2010 (Make Better Choices–MBC) [62]	Decision support; coaching algorithms. (PDA^d^)	Not specified, but possible	No	Yes, PDA allows this but not described in detail	No, although some theories mentioned
Tiwari et al 2011 [63]	Robot, dialog	Not specified in detail, but dynamic adaptation is a required feature in the design	No	No	No
Turunen et al 2011 [64] (COMPANIONS project)	Dialog; inference; automated panning	Yes, adaptive dialog, collaborative planning	No	No, but possible to include	No
Uribe et al 2011 [65]	Knowledge-based inference	Yes, reminders based on inferred mental state	No	Yes, implied in the design but not described in detail	TTM incorporated in ontology
van der Putten et al 2011 [66] (SERA project–Social Engagement with Robots and Agents)	Robot, dialog	Not mentioned	No	No	Not mentioned
Watson et al 2012 [67]	Dialog	Yes, dialog utterances tailored according to user progress with system	Not specified in detail	Not specified in detail	Relational agents

^a ^Transtheoretical model.

^b ^Motivational interviewing.

^c ^Computerized behavior intervention.

^d ^Personal digital assistant.

### Emerging Technology Themes

From Table 1, it is clear that dialog systems were used widely in interventions using active technology (19 studies). Of these, embodied conversational agents (ECA) were components of 8 studies. ECAs are visual (*embodied*) characters that can conduct conversation with a user. They are agents in the sense that they can sense and respond to their environment [68]. The agent’s environment might include a virtual game environment, the text inputs of a user, physical behavior data, or all three of these. Similarly, its responses might include conversational output, actions within the virtual environment, or effects on the real environment in the case of a robotic system [52,57,66]. Within the ECA context, motivation and empathy were central themes in 6 studies.

Ecological momentary assessment [69]) is a process of capturing the momentary experiences of participants—for example, using online diaries for the purposes of research. Not only are the participants’ environmental and behavioral circumstances recorded but so are their mental states. These can include, for example, their current goals, beliefs, mood, and emotions. Ecological momentary interventions [70]) are based on ecological momentary assessment. Levin and Levin [50] conducted a feasibility study on pain monitoring and recommended management using interactive voice response. Sorbi et al [61], in their pilot study, obtained a positive result for migraine attack prevention based on experience sampling using random mobile calls. Randomization overcomes memory bias. Another study that has relevance for the automation of ecological momentary assessment is the semantic analysis of blogs describing combat experience [48].

### Effectiveness Evaluations

Most studies (18) were prototypes or design concepts. A total of 17 were feasibility or usability studies. Only 6 were RCTs measuring effectiveness for behavior change [29,30,36,39,45,67]. All of those also included a qualitative self-report of user experience. Bickmore and Picard [29] reported mixed results for an agent-based dialog system (FitTrack), with short-term improvement in physical activity during the intervention but reduced activity after follow-up. Interacting with an agent led initially to more physical activity than the control (nonagent) for sedentary participants (45 out of 101). Users enjoyed interacting with the relational agent more than with the nonrelational agent, but there was no significant difference in physical activity between the two agent conditions (both increased during the intervention). Additionally, users found the agent’s repetitiveness to be annoying. The authors suggested that long-term interaction with the agent might prevent the drop in physical activity. Bickmore et al [30] reported positive acceptance and increased physical activity for FitTrack with older adults (n = 21) over 2 months, but there was no significant decrease in loneliness.

The study of Bickmore et al [36] involved mechanisms for reducing the repetitiveness of a dialog system and enabling it to tell stories. The effect on long-term user engagement was positive, but the effect on actual behavior was negative. The authors proposed several explanations, such as increased enjoyment during interaction inhibiting activity. Their most recent research [67] reported a positive effect (n = 70) for sustained step count over 12 weeks, but there were mixed effects for satisfaction and motivation.

The study of Consolvo et al [39] involved a personalized display of the results of an activity recognition system and reported a positive effect on actual behavior and user experience (detailed below).

Jin [45] reported a positive effect of a virtual agent on stress management self-efficacy and enjoyment (n = 60).

#### Qualitative Studies

In qualitative pilot studies (17 studies), agents with empathy and social behavior tended to be preferred over nonsocial agents. In particular, Farzanfar et al [41] found that depressed adults needed more empathy and that they found the system to be insensitive in a suicide-risk situation. There were some results that were not predicted in advance. Bickmore et al [34] studied a system to support medication adherence for mental health patients. Participants found communication with the agent to be simpler than human face-to-face communication because they could slow down the interaction and give a greater sense of control. Bickmore et al [33] reported similar effects for low health-literacy patients.

### Role of Active Technology

#### Dynamic Tailoring and User Modeling

A total of 15 studies emphasized dynamic tailoring. Of these, 10 were prototypes, 1 was an RCT [67] (detailed above), and 4 were pilot studies. The studies of Buttussi and Chittaro [38], Hayes et al [44], and Turunen et al [64] were positive but had limited evaluations. Sorbi et al [61] studied migraine prevention using ecological momentary interventions with positive acceptance, although too many calls could be annoying. Dynamic tailoring is particularly associated with user models that are generated or refined automatically. Common themes are context-aware activity recognition and intelligent reminding. Although all active assistance technology (as defined above) potentially has the capability for dynamic tailoring, we did not find this to be a major topic in most studies.

#### Interactive Education

Three studies [43,45,54] were directly concerned with health education. Hartmann et al [43] conducted a pilot study of a system to help people with asthma to participate in their own care by suggesting questions for them to ask their physicians using evidence-based decision rules. Jin’s [45] RCT tested the effect of an agent within an educational game environment on student stress management. The result was positive for self-reported stress management (n = 60).

Maier et al [53] developed a prototype for patient self-management for work-related disorders and alcohol reduction. One component was an information portal for training and health literacy, which was integrated with the Semantic Web.

#### Self-monitoring

Two studies on physical activity were concerned with accurate self-monitoring and visualization. Consolvo et al [39] conducted an RCT with positive results for a physical activity-awareness system for adults. The intervention (UbiFit) combined activity recognition with a visualization of the types of exercise that the system recognized. The visualization used a garden metaphor, where a particular type of flower represented a physical activity category (such as walking, cardiovascular, or strength). The user was awarded a flower when a physical activity was observed, eventually producing a varied garden (with a butterfly for completing the goal). Users had the opportunity to challenge and edit the system’s inferences about their activities. Using the system made a positive difference to actual exercise behavior, with the visual display having a larger positive effect. The RCT involved 28 participants, with 10 assigned to the full system, 9 assigned to activity recognition only, and 9 assigned to a manual diary with display only.

Ananthanarayan and Siek [27] reported a design concept (HealthSense) to promote teenage physical activity, based on the principles of Consolvo et al [39] but aimed specifically at young people.

### Theoretical Grounding

Behavior change models were used in 14 studies. Motivational interviewing [71] was the most widely used (8 studies). Of these, 5 were dialog systems, in which motivational interviewing was used as a general philosophy for dialog design [35,41,47,51,52]. Other behavior change theories that guided the designs were the transtheoretical model [31,35,40,47,65], self-efficacy [41,45], theory of planned behavior [28], and the health belief model [45].

#### Theoretically Grounded Ontologies

An important novel development in theoretically grounded active assistance is the incorporation of behavior change theories into the ontologies used in knowledge-based reasoning and dialog design (5 studies). The prototype in Bickmore and Sidner’s study [31] and their follow-up pilot study [35] used ontologies for automated reasoning about the behavior change process. One of these ontologies is *theory-neutral*, while another is based on the transtheoretical model, enabling reasoning about the different stages. Similarly, De Rosis et al [40], Klein et al [47], and Uribe et al [65] incorporated aspects of the transtheoretical model and other theories into an ontology for dynamic tailoring of messages, depending on the inferred mental state of the user.

## Discussion

The results show that significant research has been focused on dialog systems, ECAs, and activity recognition. There was also some work on ecological momentary intervention and intelligent context-aware prompting. The most covered health topic is physical activity. Most studies were still at an early stage, either prototype work in progress or pilot studies. Only 6 were RCTs, of which 4 were positive for behavior change and 5 were positive for acceptability.

The studies on dialog and ECA systems showed that empathy and relational behavior are significant research themes in behavior change, with many pilot studies showing preference for those features. The effect on actual behavior also tended to be positive. Too much interaction, however, might interrupt and inhibit positive health behaviors. So there is a need for careful consideration of the frequency and duration of interactive sessions in context.

Ecological momentary intervention is an opportunity for generating models from captured user experiences in the user’s own language (eg, from social networking sites) and for integrating these models with expert knowledge. Such models can include the mental states and emotions of the user, particularly if they are used in conjunction with theoretically grounded ontologies [35]. Outside the behavior change domain, recent developments in mental health management have used models of this sort [72-74].

We found relatively few studies explicitly focusing on the functions of active technology that we selected above: dynamic tailoring, interactive education, and self-monitoring. Although some interventions may have included these functions implicitly, it seems that many studies did not recognize the role of a particular technology in enabling or improving these aspects.

### Implications for Future Research

#### Links with Cognitive Science and Computer Science

Behavior change research needs to be informed by a deep understanding of algorithms and techniques that can support interventions. For this purpose, interdisciplinary collaboration with computer science and cognitive science is needed. In particular, behavior change technology has some parallels with educational technology. In educational systems, an intelligent tutor builds a model of the learner based on his or her performance and responses to questions (eg, what concepts does this person find difficult?) [75]. This model is then opened up for inspection so that the user can see how the system has represented his or her progress and misconceptions [76-80]. The open model supports the user’s self-awareness, which is also an essential component of many behavior change theories. In behavior change research, we found only 1 study [45] that was aware of educational technology research.

Making users aware of the models can draw their attention to emotions and environmental circumstances (ecology) that are associated with negative behavior outcomes. Similarly, opening up models and giving users more control may enable users to spot any serious misunderstanding by an agent or dialog system, thus avoiding the problem of users blindly following incorrect instructions. In some educational systems [81], users can persuade the automated tutor to change the model, because users are experts in their own experience (although not in the factual topics they are learning). This general principle of patient (or nonexpert) participation in health information and management is a current research topic in heath informatics [82-86].

#### Alternatives to Dialog and ECA

Most studies on active assistance technologies in behavior change are based on natural-language dialog and ECA. We did not find many alternatives to these approaches that could be used if natural language or the ECA format is not suitable or preferable. For example, users might interact with adaptive interfaces where the users’ actions are interpreted semantically as if they were dialog responses. Many of the core principles, such as model-based reasoning, activity recognition, and context-aware reminders, can be effective with different forms of interface.

#### Need for Dependable Systems

Studies on ECA and dialog systems are mostly focused on relational behavior and enjoyment of usage. If ECA systems are to be deployed in areas such as mental health and low health literacy [33,34] they will need to be validated as safe, effective, efficient, and acceptable to patients or clients. Such validation, for example in the European Union, may have to meet criteria usually applied to medical devices. This parsimonious approach makes it difficult to reflect realistic complexity—for example, the detection of emerging health problems based on subtle content of a conversation that a human expert would be able to detect. Sometimes, important decisions might be supported by ECA systems that fall outside of their validated uses. For example, errors might happen due to unexpected behavior of an algorithm. There is a need for research on making the systems robust in unplanned scenarios.

#### Need for Integrated Semantic Systems

Most studies in behavior change were focused on one or two technologies (eg, dialog and activity recognition) without specifying how the components can interact to infer further information. For example, coordination between activity recognition and content analysis of online diary entries might determine the circumstances in which relapses tend to occur, and tailor messages accordingly. Similarly, reliable automated decision making requires an interactive system to be connected with diverse specialist knowledge sources that can be requested on demand. More research is needed on how the components of an active assistance system are coordinated together and how they may be connected with the Semantic Web and other health informatics resources (eg, risk modeling).

### Limits of This Review

Articles not indexed in Google Scholar or PubMed would have been missed—most scholarly publications, however, are captured by Google Scholar. The review required the mention of “health” and “behavior change” in the articles. We did not include gray literature such as white papers and unpublished reports. We selected the date range (2005–2012) to focus on recent developments, but this may also have excluded innovative earlier work.

The review required specific mention of a key technology. There may be some interventions that use active technologies, but the studies did not mention this. Similarly, some studies mentioning only general intelligent technology were excluded from the full-text review because they could not be categorized. This may be a limitation because included studies need to involve significant interdisciplinary communication between technology specialists and health specialists. On the other hand, it may be a strength, as such communication is important for understanding a particular technology in context.

Since we limited the search to behavior change, it is also possible that many of the technologies are being applied in other areas of health informatics. For example, we found some prototypes early in the date range (2006–2007) but found no subsequent study relating to behavior change. In these cases, citation searching sometimes revealed further development of the techniques and algorithms, but no application in the health domain.

### Conclusion

The potential of active technologies for dynamic and unbiased information processing is not being fully exploited in current health behavior change research. Most research has focused on specialist areas, such as dialog and ECA systems, and has been largely restricted to the study of persuasive dialog in respect of relational behavior and motivation of behavior change.

In addition to the potential benefits of active technologies, there is a need for a thorough understanding of the potential risks. Expected benefits such as that of dynamic tailoring of the content and presentation of information can be measured using established evaluation methods (eg, [87,88]). Risks such as misinformation due to the unexpected behavior of an algorithm may be more difficult to uncover. It is important to study realistically complex scenarios of the uses of active assistance systems. Such studies need to reveal how the system components interact to produce information, and how these components might in turn interact with wider systems such as the Semantic Web, clinical records, and personal health records. Wider still, we note that many health behaviors are socially mediated; therefore, active assistance research needs to bridge cognitive and sociotechnical aspects in order to deliver maximum public health benefit.

To exploit the full potential of active assistance technology, health behavior change researchers need a deep understanding of how the different components of information systems might change the intervention—its safety, effectiveness, efficiency, and acceptability. This requires more collaboration between disciplines such as health psychology, computer science, cognitive science, health informatics, medical sociology, and public health and health promotion.

## Abbreviations

ECA: embodied conversational agent
RCT: randomized controlled trial
